# Aberrant Structural Connectivity of the Triple Network System in Borderline Personality Disorder Is Associated with Behavioral Dysregulation

**DOI:** 10.3390/jcm11071757

**Published:** 2022-03-22

**Authors:** Giulia Quattrini, Laura Rosa Magni, Mariangela Lanfredi, Laura Pedrini, Antonino Carcione, Ilaria Riccardi, Daniele Corbo, Roberto Gasparotti, Roberta Rossi, Michela Pievani, the CLIMAMITHE Study Group

**Affiliations:** 1 Laboratory Alzheimer’s Neuroimaging and Epidemiology, IRCCS Istituto Centro San Giovanni di Dio Fatebenefratelli, 25125 Brescia, Italy; gquattrini@fatebenefratelli.eu (G.Q.); mpievani@fatebenefratelli.eu (M.P.); 2 Department of Molecular and Translational Medicine, University of Brescia, 25123 Brescia, Italy; 3 Unit of Psychiatry, IRCCS Istituto Centro San Giovanni di Dio Fatebenefratelli, 25125 Brescia, Italy; laurarosa.magni@gmail.com (L.R.M.); mlanfredi@fatebenefratelli.eu (M.L.); lpedrini@fatebenefratelli.eu (L.P.); 4 Third Centre of Cognitive Psychotherapy, 00161 Rome, Italy; nino.carcione@gmail.com (A.C.); ila.riccardi@gmail.com (I.R.); 5 Italian School of Cognitive Psychotherapy (SICC), 00185 Rome, Italy; 6 Neuroradiology Unit, Department of Medical and Surgical Specialties, Radiological Sciences and Public Health, University of Brescia and ASST Spedali Civili Hospital, 25123 Brescia, Italy; daniele.corbo@unibs.it (D.C.); roberto.gasparotti@unibs.it (R.G.)

**Keywords:** borderline personality disorder, triple network, DTI, structural connectivity, mean diffusivity, behavioral dysregulation

## Abstract

Background: Core symptoms of Borderline Personality Disorder (BPD) are associated to aberrant connectivity of the triple network system (salience network [SN], default mode network [DMN], executive control network [ECN]). While functional abnormalities are widely reported, structural connectivity (SC) and anatomical changes have not yet been investigated. Here, we explored the triple network’s SC, structure, and its association with BPD clinical features. Methods: A total of 60 BPD and 26 healthy controls (HC) underwent a multidomain neuropsychological and multimodal MRI (diffusion- and T1-weighted imaging) assessment. Metrics (fractional anisotropy [FA], mean diffusivity [MD], cortical thickness) were extracted from SN, DMN, ECN (triple network), and visual network (control network) using established atlases. Multivariate general linear models were conducted to assess group differences in metrics and associations with clinical features. Results: Patients showed increased MD in the anterior SN, dorsal DMN, and right ECN compared to HC. Diffusivity increases were more pronounced in patients with higher behavioral dysregulation, i.e., suicidal attempting, self-harm, and aggressiveness. No differences were detected in network structure. Conclusions: These results indicate that the triple network system is impaired in BPD at the microstructural level. The preferential involvement of anterior and right-lateralized subsystems and their clinical association suggests that these abnormalities could contribute to behavioral dysregulation.

## 1. Introduction

Borderline Personality Disorder (BPD) is a complex psychiatric disease defined by instability in personal relationships, emotional dysregulation (e.g., inappropriate intense anger or ongoing feelings of emptiness, disturbed self-image, fear of being abandoned), and behavioral dysregulation (impulsive and aggressive behaviors, repeated self-harming and suicide attempts) [1]. BPD symptoms have been proposed to be associated to the impairment of core emotional and cognitive networks, i.e., the salience network (SN), the default mode network (DMN), and the executive control network (ECN), according to the “triple network model” [2,3]. The SN plays a critical role in emotion regulation by detecting relevant stimuli and selecting appropriate behavioral responses, and in switching between the DMN and ECN. This circuitry includes core regions for emotional processes such as the insula and the amygdala. The DMN, which includes the precuneus/posterior cingulate cortex, medial prefrontal cortex, hippocampus, and lateral temporal, parietal and frontal cortex cortices, is typically active during rest and is involved in self-related cognitive processes, self-monitoring, autobiographical memory, and social functions. Finally, the ECN includes the bilateral dorsolateral frontal and parietal cortices and is associated to cognitive and executive control processes during goal-directed behavior and attention-demanding tasks.

The hypothesis of a triple network’s model dysfunction in BPD is supported by resting-state functional MRI studies reporting SN, DMN, and ECN functional connectivity (FC) alterations in patients compared to healthy controls (HC). Aberrant FC in the SN has been associated with emotional and social behavior dysregulations, lower metacognitive abilities, altered reward and salience processing, and impaired self-relevance evaluation [4,5,6,7,8,9,10], while in the DMN have been associated to emotional dysregulation, including pain processing [4,11,12,13]. Finally, FC alterations in the ECN have been associated to impulse dyscontrol [14,15].

Less clear is whether these networks are structurally impaired in BPD. Core cognitive processes depend on both functional and structural integrity of the network’ nodes and anatomical connections. While FC is strictly associated to structural connectivity (SC), they are not equivalent and reflect distinct brain properties: FC denotes the temporal synchronization between neurons, while SC reflects their anatomical connections (white matter (WM) fibers) [16,17,18]. Thus, while FC provides information on cortical desynchronization, SC can clarify the anatomical underpinning of impairment. Anatomical connections can be assessed with diffusion tensor imaging (DTI), a neuroimaging technique providing information about microstructural organization of WM by measuring water diffusion. Traditionally, four indices of diffusion are assessed using diffusion tensor imaging (DTI): fractional anisotropy (FA) and mean (MD), radial (RD), and axial (AD) diffusivities [19,20]. While FA and MD are general measures of microstructural organization sensitive to pathological and developmental processes (e.g., demyelination, axonal loss, inflammation, axonal growth), RD and AD refer to the preferential direction of diffusion (i.e., perpendicular and parallel to fiber orientation, respectively) and are potentially more specific markers of axonal demyelination and damage, respectively [20,21]. Previous DTI studies in BPD consistently reported WM alterations (lower FA, increased MD, RD, and AD) in associative tracts connecting the frontal cortex to other cortical regions, such as the genu of the corpus callosum, frontolimbic (e.g., cingulum and fornix), frontoparietal (e.g., superior longitudinal fasciculus) and fronto-occipital (e.g., inferior longitudinal fasciculus) tracts. Microstructural changes in these tracts were also associated to core symptoms, such as self-harm and suicidal behavior, affective instability, anger expression, avoidance of abandonment, and anxiety [22,23,24,25,26,27,28,29,30,31,32]. However, to the best of our knowledge, to date no study has specifically assessed microstructural abnormalities in the WM tracts connecting the nodes of the triple network model.

In addition to SC impairment, abnormalities in structure/anatomy can also affect network’s dysfunction. Brain structural integrity can be assessed with cortical thickness or volumetric assessment from structural MRI. Previous MRI studies in BPD reported altered cortical thickness in areas potentially part of the triple network system (e.g., parahippocampal cortex, precuneus, and posterior cingulate cortex for the DMN, anterior cingulate cortex and insula for the SN, dorsolateral prefrontal cortex and parietal cortex for the ECN) [33,34,35,36,37]. However, a direct assessment of the triple network system structural integrity is lacking.

The first aim of this study was to assess whether BPD patients show WM microstructural impairment of the triple network system. Moreover, to test the specificity of triple network impairment, we included the visual network as a control network as no study has never reported any impairment of this network in BPD. The second aim was to assess if the triple network system is affected at the structural level. Third, we explored whether microstructural and anatomical impairment of the three networks was associated with clinical measures.

## 2. Materials and Methods

### 2.1. Subjects

A total of 60 BPD patients and 26 HC were enrolled between December 2015 and November 2018, in the context of the CLIMAMITHE study [38]. All data included in the present study were collected at the baseline timepoint. Exclusion and inclusion criteria, as well as the clinical assessment have been described elsewhere [38]. Briefly, the BPD diagnosis was confirmed through the Structured Clinical Interview for DSM-IV (SCID I and II), while HC were volunteers with no ascertained psychiatric diagnosis and family history for BPD. The clinical evaluation was conducted following a multidimensional approach and included the following scales: the Zanarini Rating Scale for Borderline Personality Disorder (ZAN-BPD, to evaluate the severity of BPD symptomatology), the Difficulties in Emotion Regulation Scale (DERS, for problems in the emotion regulation), the Barratt Impulsiveness Scale (BIS-11, to assess the level of impulsivity), Symptoms Check List-90-R (SCL-90-R, for the assessment of general psychopathology), the State-Trait Anger Expression Inventory (STAXI-2, to estimate the intensity of angry feelings, experience, control, and expression), and the Childhood Trauma Questionnaire (CTQ, to retrospectively assess childhood abuse and maltreatment). Patients also underwent the Metacognition Assessment Interview (MAI) to investigate metacognitive abilities.

All participants provided written informed consent according to the declaration of Helsinki. The study was approved by the local Ethics Committee of the IRCCS Fatebenefratelli (Comitato Etico delle Istituzioni Ospedaliere Cattoliche).

### 2.2. MRI Acquisition

MRI sequences were acquired on a 3 Tesla scanner equipped with a 64 channels RF head coil (Skyra Siemens, Erlangen, Germany) at the Neuroradiology Unit of the Spedali Civili Hospital (Brescia, Italy). DTI was acquired along sixty-four non-collinear gradient directions (b = 1000 s/mm^2^) and five non-weighted directions (b = 0 s/mm^2^) using an axial spin-echo EPI sequence (TR = 8300 ms, TE = 75 ms, voxel size = 2.0 × 2.0 × 2.0, FoV = 224 mm, slice thickness = 2 mm). In addition, five non-weighted EPI scans (b = 0 s/mm^2^) were collected with reversed phase-encoding blips for distortion correction.

Structural 3D T1-weighted images were collected with the following parameters: TR = 2300 ms; TE = 2 ms; flip angle = 9; spatial resolution = 1 mm isotropic, 176 sagittal slices.

### 2.3. MRI Preprocessing

#### 2.3.1. Diffusion Tensor Images (DTI) Analysis

One scan (BPD) was excluded from the analysis due to a technical problem during the DTI acquisition. Data preprocessing was performed using the FMRIB’s Diffusion Toolbox (FDT, http://fsl.fmrib.ox.ac.uk/fsl/fslwiki/FDT, accessed on 2 February 2022), part of the FMRIB’s Software Library (FSL, http://www.fmrib.ox.ac.uk/fsl/, accessed on 2 February 2022), version 5.0.9. First, the two non-weighted EPI images were used to estimate the susceptibility-induced off resonance field as implemented in the *top-up* tool of FSL [39] (https://fsl.fmrib.ox.ac.uk/fsl/fslwiki/topup, accessed on 2 February 2022). Then, the DTI sequences were corrected for eddy current induced distortions and subject movements using FSL’s *eddy* [40]. For each subject, the diffusion tensor was estimated on the eddy-corrected data with DTIfit (FDT toolbox) and the diffusion maps (FA, MD, RD, and AD) were created. A visual quality check was carried out on all the output images.

The WM tracts connecting the nodes of the SN (anterior and posterior components), the DMN (separately for dorsal and ventral subdivisions), the ECN (segregated into left and right components), and the visual network (VIS; control network) were identified using probabilistic fMRI-guided atlases [41,42]. For each of the above 7 networks, the corresponding WM probability map was thresholded at 5% to exclude voxels with a low probability of being part of the network and then binarized. The WM probability maps, which are provided in MNI space, were then warped in individual diffusion native space as follows. First, the FSL Tract-Based Spatial Statistics (TBSS) tool [43] was used to estimate the non-linear transformations bringing the DTI images into MNI space. To this aim, TBSS first selects the most representative subject in the sample by non-linearly aligning every FA image to every other one using the fMRIB’s Nonlinear Image Registration Tool (FNIRT) [44]. Then, the target image is automatically affinely-aligned into MNI standard space using the FMRIB’s Linear Image Registration Tool (FLIRT) [45]. For each subject, the non-linear and the affine transformations are combined into a single transform and the inverse warps (bringing MNI to native space) were calculated. Finally, these inverse transformations were used to project the network WM maps to native diffusion space. The network maps were thresholded at a level of 90% and binarized to obtain the Regions of Interest (ROIs) (Figure S1). These ROIs were overlaid to each subject diffusion map (FA, MD, RD, and AD), and the mean values were then extracted.

#### 2.3.2. Structural 3D T1-Weighted Images Analysis

All 3D T1-weighted MRI scans underwent a visual quality check according to a published rating system [46], and 1 scan was excluded from BPD sample due to motion artifacts. Images were processed using the FreeSurfer v6.0 cross-sectional standard pipeline (https://surfer.nmr.mgh.harvard.edu/, accessed on 2 February 2022) [47]. The automated procedure includes motion and non-uniformity corrections, skull stripping, linear and non-linear Talairach transformations from native to MNI305 standard space, intensity normalization, white matter and deep structures segmentation, gray and white matter boundary tessellation, topology correction, surface deformation and definition based to the intensity gradients. Several deformable procedures (surface inflation and registration to a spherical atlas, and cortex parcellation) were then implemented to create surface-based data. Each output was visually inspected, and 6 subjects (5 BPD and 1 HC) were excluded due to processing failures. The Yeo networks atlas [48], a cortical parcellation based on intrinsic functional connectivity, was used to measure the mean cortical thickness within each network. The atlas was overlaid on FreeSurfer surface-based data with the *mri_surf2surf* function. Network 4 (ventral attention network, for the SN), network 7 (DMN), network 6 (ECN), and network 1 (VIS) were selected (Figure S2). For each network, cortical thickness was computed (*mris_anatomical_stats*). For the DMN, SN, and VIS, cortical thickness values were averaged between left and right hemispheres, while the left ECN (LECN) and right ECN (RECN) were considered separately.

## 3. Statistical Analysis

Statistical analyses were conducted using IBM SPSS Statistics for Windows, version 23.

Group differences were assessed using the two-sample *t*-test or the Mann–Whitney *U* test, based on the data distribution (for continuous variable), and the Pearson’s chi-square test (for categorical variables).

For the first aim of this study, two multivariate General Linear Models (GLMs) were conducted to assess group differences in diffusivity of the 7 selected WM networks. In a first-level analysis, between-groups differences in FA and MD values were assessed. Then, a second-level analysis was conducted including RD and AD values only from those networks for which a between-group difference in MD was detected, to further explore the possible mechanism underlying microstructural alterations.

For the second aim, a multivariate GLM was conducted to assess group differences in cortical thickness within the DMN, SN, LECN, RECN, and VIS.

Finally, for the third aim, the relationship between the triple network’s features and BPD (i) clinical features and (ii) behavioral dysregulation was explored. To assess the association with BPD clinical features, the Pearson’s correlation coefficient or the Spearman’s rank correlation coefficient (based on data distribution) were conducted including DTI and/or cortical thickness and the score at clinical scales (ZAN-BPD, DERS, BIS-11, SCL-90, STAXI-2, CTQ, and MAI). To assess associations with behavioral dysregulation, all subjects were classified according to the presence/absence of suicide attempts, self-harm, and hetero-directed aggressive behaviors, and six multivariate GLMs were performed (i.e., first and second-level analyses for suicide attempts, self-harming, and aggressive behavior). FA and MD were included in the first-level analysis, while RD and AD only from those networks for which a between-group difference was detected were then entered in the second-level analysis. The relationship between the triple network’s features and BPD clinical features and behavioral dysregulation was explored only for DTI and/or cortical thickness measures that emerged as different between groups (from aim 1).

All multivariate GLMs were corrected for multiple comparisons using the Bonferroni correction.

Significance (*p* value) was set to *p* < 0.050 for all statistical tests.

## 4. Results

### 4.1. Demographical and Clinical Features of Participants

Table 1 summarizes the main demographic and clinical features of BPD and HC. Groups were comparable for age and sex, while differed for education. Patients had a history of disease of 11.8 ± 7.6 years on average, and a history of abuse/dependence of alcohol (22.6 ± 45.0 lifetime months) and/or substances (25.6 ± 52.8 lifetime months). Most patients reported familiarity with psychiatric disease (78%) and traumatic experiences (80%), while half reported behavioral dysregulation in the form of self-harm (50%), aggressiveness (53%), or suicide attempts (48%). Finally, a proportion of patients was on pharmacotherapy, including mood stabilizers (35%), benzodiazepines (35%), atypical antipsychotics (31%), and SSRI antidepressants (31%). Few patients received SNRI (1%), multimodal (5%), or tricyclic (3%) antidepressants, hypnotics (3%), and typical antipsychotics (2%).

### 4.2. Mean Diffusivity Is Increased in the Triple Network System of BPD

**Figure 1:**
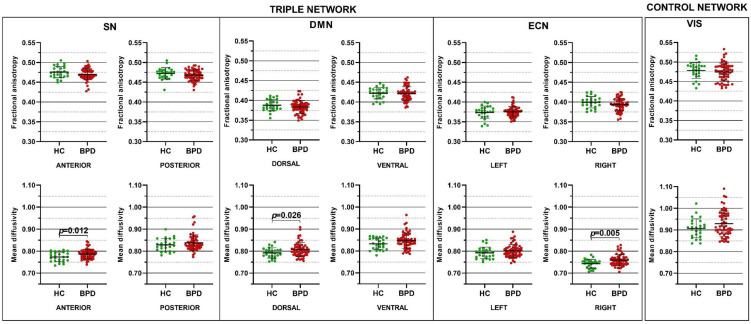
Differences in fractional anisotropy and mean diffusivity of the salience network (SN), default mode network (DMN), executive control network (ECN), and visual network (VIS) in Borderline Personality Disorder (BPD) compared to healthy control (HC). Each dot represents a subject, horizontal and vertical bars denote the mean and the standard deviation, respectively. *p* values denote the statistical significance, corrected for multiple comparisons using the Bonferroni method.

In the first-level analysis, the multivariate GLM revealed MD increases for the dorsal DMN (*p* = 0.026), anterior SN (*p* = 0.012), and RECN (*p* = 0.005) in BPD compared to HC (Figure 1). A trend for statistical significance also emerged for the MD in the ventral DMN (*p* = 0.057). No difference was detected for FA nor for DTI metrics of VIS (Figure 1).

In the second-level analysis, the multivariate GLM revealed both RD and AD increases in BPD compared to HC in the dorsal DMN (*p* = 0.036 and *p* = 0.030, respectively), anterior SN (*p* = 0.012), and RECN (*p* = 0.007, and *p* = 0.014, respectively). A trend for statistical significance also emerged for the AD in the anterior SN (*p* = 0.052).

Finally, the multivariate GLM did not reveal any group differences for networks’ cortical thickness (*p* > 0.050).

### 4.3. WM Alterations Are Associated with Behavioral Dysregulation in BPD

In patients, correlation analyses did not reveal any significant association between diffusivity abnormalities (MD, RD, and AD from anterior SN, dorsal DMN, and RECN) and clinical features (all *p* > 0.050).

When subjects were stratified according to behavioral dysregulation, significant group differences emerged for suicide attempts, self-harm and aggressive behaviors.

**Figure 2:**
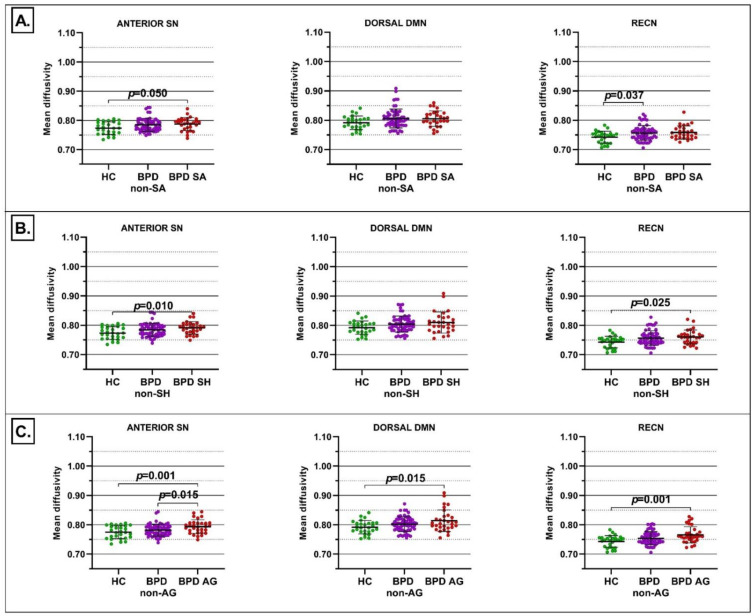
Differences in mean diffusivity of the anterior SN, dorsal DMN, and RECN between BPD patients with suicide attempts (BPD SA) and without suicide attempts (BPD non-SA) and HC (panel (**A**)), between BPD patients with self-harm (BPD SH) and BPD without self-harm (BPD non-SH) and HC (panel (**B**)), and between BPD patients with aggressive behaviors (BPD AG) and BPD without aggressive behaviors (BPD non-AG) and HC (panel (**C**)). Each dot represents a subject, horizontal and vertical bars denote the mean and the standard deviation, respectively. *p* values denote the statistical significance, corrected for multiple comparisons using the Bonferroni method.

For suicide attempts, in the first-level analysis the multivariate GLM was statistically significant for MD in the anterior SN (*F* = 3.385, *p* = 0.039) and RECN (*F* = 4.041, *p* = 0.021). No statistical significance was detected for FA nor for DTI metrics of DMN and VIS. Pairwise comparison revealed higher MD in the anterior SN in BPD with suicide attempts episodes compared to HC (*p* = 0.050), while higher MD was reported in the RECN in BPD without suicide attempts episodes compared to HC (*p* = 0.037) (Figure 2A). The second-level analysis confirmed the involvement of both RD and AD in the above comparisons (*p* < 0.050).

For self-harming behaviors, in the first-level analysis the multivariate GLM was statistically significant for MD in the anterior SN (*F* = 4.583, *p* = 0.013) and RECN (*F* = 4.153, *p* = 0.019). No statistical significance was detected for FA nor for DTI metrics of the DMN and VIS. Pairwise comparison revealed higher MD in the anterior SN (*p* = 0.010) and RECN (*p* = 0.025) in BPD with self-harming behaviors compared to HC (Figure 2B). The second-level analysis confirmed the involvement of RD and AD in the above comparisons (*p* < 0.050).

Finally, for aggressive behaviors, in the first-level analysis the multivariate GLM was statistically significant for MD in the dorsal DMN (*F* = 4.416, *p* = 0.015), anterior SN (*F* = 7.576, *p* < 0.001), and RECN (*F* = 7.050, *p* = 0.002). No statistical significance was detected for FA nor for DTI metrics of VIS. Pairwise comparison revealed higher MD in the dorsal DMN (*p* = 0.015), anterior SN (*p* = 0.001), and RECN (*p* = 0.001) in BPD with aggressive behavior compared to HC (Figure 2C). Higher MD was also reported in the anterior SN (*p* = 0.015) in BPD with aggressive behavior compared to BPD without aggressive behavior (Figure 2). The second-level analysis confirmed the simultaneous involvement of RD and AD in all the above comparisons (*p* < 0.050).

## 5. Discussion

Abnormalities in the triple network’s FC have been widely documented in BPD [4,5,6,7,8,9,10,11,12,13,14,15]. Our results extend this evidence showing for the first time that triple network’s impairment in BPD also affects WM microstructure (i.e., SC), while the circuits’ morphology is spared. The first-level analysis highlighted the selective increase of MD in BPD compared to HC. The second-level analysis clarified the potential underling mechanism: WM alterations were probably related to axonal demyelination (increased RD) and axonal damage (increased AD) [20]. This dysconnectivity pattern was also associated to behavioral dysregulation in BPD.

The first aim of our study was to test the hypothesis of an SC impairment of the triple network system in BPD. Our results confirmed this hypothesis, as we found WM abnormalities in the DMN, SN, and ECN. Moreover, these abnormalities did not affect the control network (VIS), supporting the hypothesis of a specific involvement of the triple network’s system in the disease etiology. Our data highlighted a frontal (anterior SN and dorsal DMN) and right-lateralized (RECN) pattern of WM fibers alterations in patients. These results are in line with previous DTI studies reporting abnormalities predominantly in frontal, frontolimbic, and frontoparietal tracts in BPD [22,23,24,25,26,27,28,29,30,31,32]. However, these studies did not specifically investigate the tracts connecting nodes of the triple network system, thus we can only speculate on whether these pathways are congruent with our data. A potential explanation for this impaired architecture might involve brain structural development. Previous studies on brain development showed a posterior-to-anterior and inferior-to-superior pattern of increasing FA and decreasing MD, the frontal and the temporal areas being the last to complete the WM myelination and axonal maturation process, FA and MD *plateauing* in the third decade of life [49,50,51]. Moreover, the trajectory of axonal development is known to be influenced by genetic and environmental factors, including child neglect and traumatic experiences [49,50]. In our sample, the increased MD, RD, and AD in frontal and temporal brain regions (i.e., dorsal DMN, anterior SN, and RECN) may indicate reduced axonal and/or myelin development due to impaired maturation process. This hypothesis is supported by a previous DTI study reporting microstructural alterations in frontolimbic tracts in treatment-naïve young adult with BPD compared to HC [25]. Moreover, in our sample the majority of patients had familiarity for psychiatric diseases (78%) and experienced traumatic events (80%), thus we speculate that genetic and/or environmental variables could also have influenced axonal maturation and/or myelination in the triple network system of BPD.

The second aim of this study was to assess if the triple network’s structural impairment also involved network’s anatomy/morphology. Our results showed that abnormalities were limited to SC, suggesting that BPD might be a disease mostly related to networks’ disconnection than morphological abnormalities, and that the nodes of this system may not show relevant changes at the structural level until advances stages. This evidence could have potential consequences for therapeutic interventions. In this sense, recent randomized controlled trials reported beneficial effects of non-invasive brain stimulation in BPD, reducing impulsivity and aggression and improving the cognitive control [52,53,54,55]. As non-invasive stimulation exerts effect both on synaptic plasticity and axonal pathways [56] and its efficacy is probably greater when plasticity is not compromised, the relative sparing of the triple network system morphology might support the efficacy of this treatment.

The third aim of this study was to investigate the association between the triple network’s WM microstructural alterations and clinical features. Our data confirmed that microstructural abnormalities in the triple network system were associated with behavioral dysregulation. Suicidal attempts, self-harm, and aggressiveness toward others are related to emotional instability and impulsivity in BPD [57,58,59]. Our data pointed to a specific involvement of the anterior subsystem of the SN, which is anchored to the insula, the core hub playing a critical role in the regulation of the engagement/disengagement of the DMN and ECN [2,60,61]. We speculate that alterations in the anterior SN could impair salience attribution to external and/or internal stimuli and this may in turn contribute to the emotional instability underling BPD impulsive behaviors [62,63]. As concerns the ECN, SC impairments emerged in BPD non-attempters and in BPD with self-harm and aggressive behaviors. Emotional stimuli processing, irrespectively of the valence, generally engage the right hemisphere, where the emotional and attentional cognitive systems interact to select the correct response to the context [64]. In our sample, the right lateralization of SC impairment probably accounts for a general impairment of emotion processing in BPD non-attempters [14,15]. On the other hand, patients with self-harm and aggressive behaviors showed both SN and RECN impairment, suggesting that self- and hetero-directed aggressive behaviors could be also related to the joint impairment of attention switching (anterior SN) and behavioral response selection to emotional stimuli (RECN) processes [61,64]. The compromission of the SN, which plays a coordination role within the triple network system [2], might account for the difficulty in selecting the correct behavior response to internal/external stimuli in BPD. Aggressiveness towards others was also related to anterior DMN disconnections. In line, a previous FC study reported the association between BPD aggressiveness and connectivity of a single, widespread network encompassing DMN (posterior cingulate cortex), SN (insula), and ECN (dorsolateral prefrontal cortex) hubs [65]. In this sense, the triple network’s system impairment seems to be more pervasive in hetero- than in self-aggressive behaviors.

This study has several strengths. First, to the best of our knowledge, this is the first assessment of SC in the triple network system in BPD. While previous studies reported WM impairment in pathways possibly part of these circuitries, this is the first direct investigation using a network-based approach. Second, this is also the first study to directly assess this system morphology in BPD. Finally, the large BPD sample allowed the stratification according to behavioral features.

This study also has limitations. Patients often reported a history of abuse or dependence of alcohol and/or substance, and the effect of these conditions on WM integrity is well documented [66]. However, this is a common feature of BPD and the exclusion of patients with a past dependence would have limited the generalizability of the results. We did not include these features as covariates in the GLM due to collinearity issues. As concerns methodology, we used an fMRI-guided atlas to detect WM tracts connecting networks nodes. An optimal approach would use the fMRI data of the study sample to guide fibers extraction. Future studies, collecting both DTI and resting-state fMRI sequences were, will enable to assess both SC and FC changes in the triple network system and to investigate their reciprocal relationship.

## 6. Conclusions

Our results confirmed the impairment of the triple network system connectivity in BPD and indicated a specific anterior and right-lateralized pattern of microstructural changes. Importantly, this pattern was related to behavioral dysregulation, representing a potential marker of the disease and a possible target for future non-invasive treatments using brain stimulation.

## Figures and Tables

**Table 1:** Demographic and clinical features of Borderline Personality Disorder (BPD, *n* = 59) and healthy controls (HC, *n* = 26). Values are reported as mean (*M*) ± standard deviation (*SD*) or percentage (%). *U* denotes the Mann–Whitney U test value, *t* denotes the two-sample *t*-test value, *X*^2^ denotes the Pearson’s chi-square test value, *df* denotes the degrees freedom, while *p* denotes their statistical significance (set to *p* < 0.050). Significant results are reported in **bold**.

Demographic Features	BPD	HC	Test Value (*df*)	*p* Value
Age, years	29.1 ± 7.7	28.9 ± 6.9	*U* = 759.5	0.943
Sex, females %	88%	81%	*X*^2^(1) = 0.808	0.369
Education, years	12.9 ± 3.1	17.3 ± 2.2	*U* = 227.0	**<0.001**
**Clinical features**				
SCL-90-R	181.0 ± 66.0	24.8 ± 19.3	*U* = 4.0	**<0.001**
BIS-11	74.6 ± 12.0	54.8 ± 8.8	*t*(81) = 7.422	**<0.001**
DERS	125.3 ± 22.9	64.6 ± 11.7	*U* = 12.0	**<0.001**
ZAN-BPD	16.5 ± 5.1	2.1 ± 1.8	*U* = 1.50	**<0.001**
STAXI-2				
Anger/state	23.9 ± 10.2	15.3 ± 0.6	*U* = 218.5	**<0.001**
Anger/trait	7.8 ± 10.6	2.9 ± 4.5	*U* = 344.0	**<0.001**
Anger expression/out	20.8 ± 4.7	14.4 ± 3.8	*U* = 199.5	**<0.001**
Anger expression/in	23.0 ± 4.6	16.3 ± 3.7	*t*(81) = 6.430	**<0.001**
Anger control/out	14.3 ± 4.2	19.9 ± 4.1	*t*(81) = −5.570	**<0.001**
Anger control/in	16.8 ± 4.4	23.3 ± 4.7	*t*(81) = −6.155	**<0.001**
Anger expression/index	60.1 ± 11.4	35.6 ± 11.9	*t*(81) = 9.098	**<0.001**
CTQ	62.9 ± 13.9	44.8 ± 3.5	*U* = 82.5	**<0.001**
MAI	32.5 ± 6.8	-	-	-

Abbreviations: SCL-90-R, Symptoms Check List-90-R; BIS-11, Barratt Impulsiveness Scale; DERS, Difficulties in Emotion Regulation Scale; ZAN-BPD, Zanarini Rating Scale for Borderline Personality Disorder; STAXI-2, State-Trait Anger Expression Inventory; CTQ, Childhood Trauma Questionnaire; MAI, the Metacognition Assessment Interview.

## Data Availability

The data supporting the conclusions of this study can be found in Mendeley Data: doi:10.17632/syr3j7fh3p.1.

## Supplementary Materials

The following supporting information can be downloaded at: https://www.mdpi.com/article/10.3390/jcm11071757/s1, Figure S1. Example of binary masks of WM tracts underling each network, overlaid to the structural 3D T1-weighted native image of a healthy control. Figure S2. Cortical areas included within each network, overlaid to the structural 3D T1-weighted native image of a healthy control. Table S1. Summary table of WM tracts underling each network, based on the Johns Hopkins University (JHU) atlas [67].
