# Supplementary material for: Aberrant Structural Connectivity of the Triple Network System in Borderline Personality Disorder Is Associated with Behavioral Dysregulation
Source: J Clin Med. 2022 Mar 22;11(7):1757. doi: 10.3390/jcm11071757 (PMC8999477; doi:10.3390/jcm11071757)
Supplement: Supplementary file 1 [file jcm-11-01757-s001.zip › jcm-1629621-supplementary.pdf]

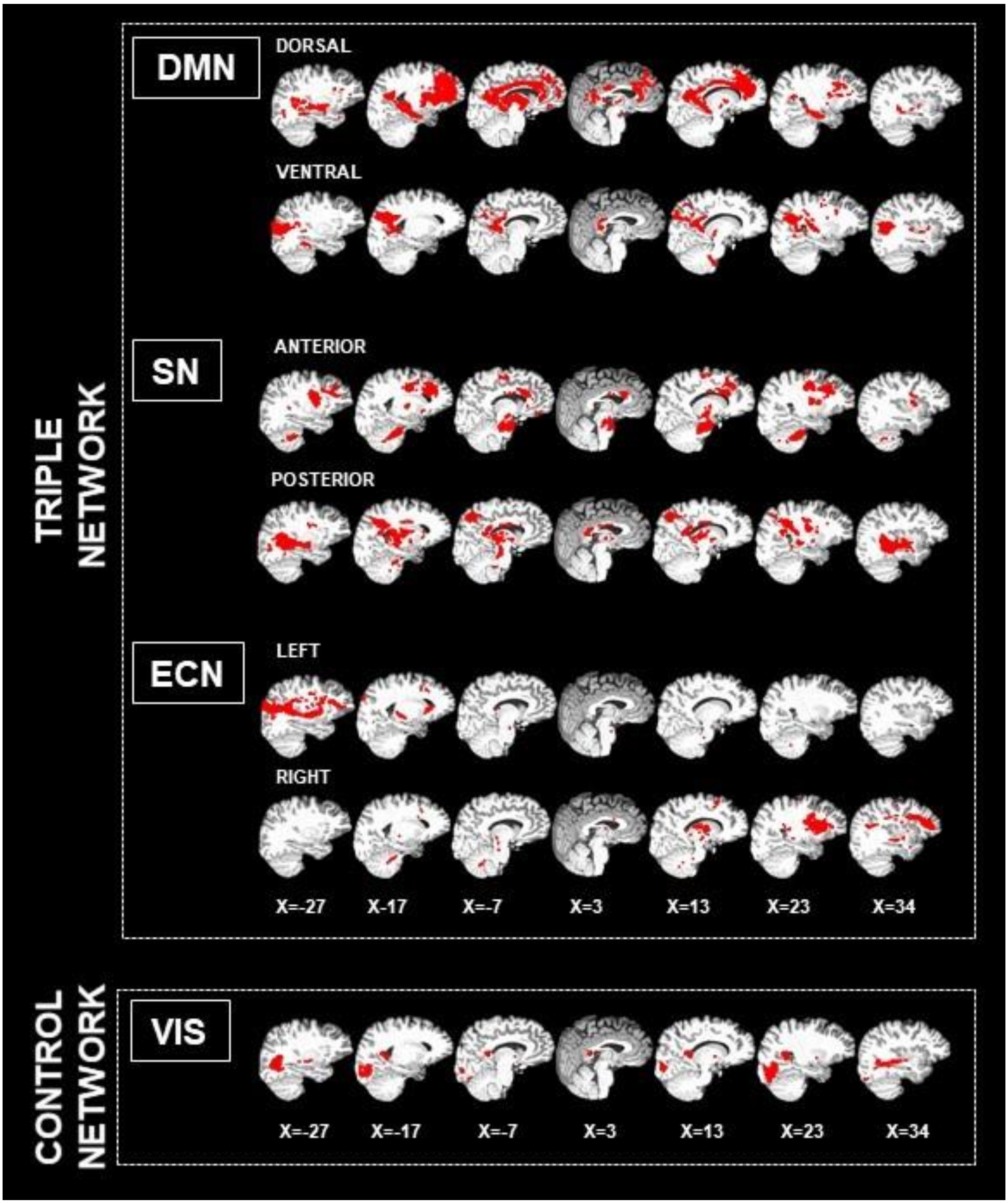

Figure S1. Example of binary masks of WM tracts underling each network, overlaid to the structural 3D T1-weighted native image of a healthy control.

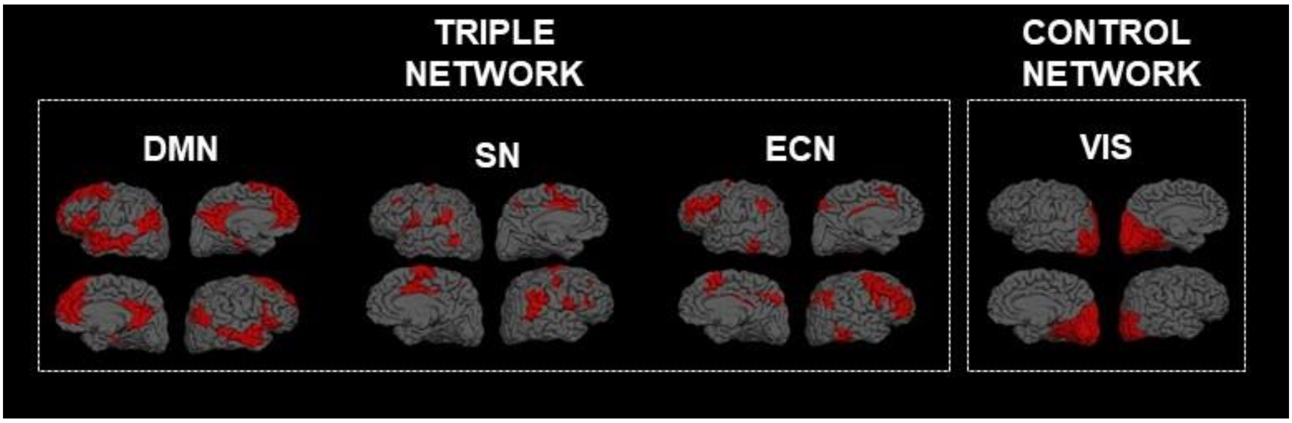

Figure S2. Cortical areas included within each network, overlaid to the structural 3D T1-weighted native image of a healthy control.

Table S1. Summary table of WM tracts underling each network, based on the Johns Hopkins University (JHU) atlas [67].

	**DMN**		**SN**		**ECN**		**VIS**
**WM tract**	**Dorsal mm^3**	**Ventral mm^3**	**Anterior mm^3**	**Posterior mm^3**	**Left mm^3**	**Right mm^3**	**mm^3**
Corpus callosum							
Genu	3208	18	3019	75	0	147	0
Body	1416	790	1414	4250	0	116	23
Splenium	4083	6305	95	5434	0	93	4943
Corona radiate							
Anterior	6108	217	2451	328	1527	2861	0
Posterior	845	2231	66	4154	502	140	289
Superior	33	255	3050	2722	130	281	0
Cerebellar peduncle	512	39	1537	475	177	374	0
Superior	0	76	45	285	0	34	0
Mid	0	41	8961	1080	254	779	0
Inferior	0	329	14	22	0	6	0
Cingulum							
Gyrus	4863	795	636	30	0	0	0
Hippocampus	2121	78	0	231	0	0	0
Corticospinal tract	5	0	845	231	86	16	0
Medial lemniscus	0	1	37	109	0	0	0
External capsule	939	345	1004	1174	1916	877	121
Fornix	114	0	0	35	0	26	0
Stria terminalis	504	0	0	914	0	56	0
Limb of the internal capsule							
Anterior	2938	81	355	968	166	290	0
Posterior	616	179	1278	2371	89	344	0
Retrolenticular	753	827	8	2393	1237	299	

Posterior thalamic radiation	999	210	28	3163	428	0	3231
Sagittal stratum *	671	64	0	2718	0	180	134
Superior front-occipital fasciculus	7	23	0	70	0	177	0
Superior longitudinal fasciculus	15	52	272	78	1813	745	0
Tapetum	36	0	0	551	0	5	103
Uncinate fasciculus	32	0	0	29	0	13	0
Pontine crossing tract	2	0	573	10	0	5	0

* Includes inferior longitudinal fasciculus and inferior fronto-occipital fasciculus.
